# ELECTROCONVULSIVE THERAPY FOR TREATMENT OF TACTILE HALLUCINATIONS

**DOI:** 10.1093/geroni/igad104.2438

**Published:** 2023-12-21

**Authors:** Megan Kummerlowe, Kierstin Utter, Lapid Maria

**Affiliations:** Mayo Clinic, Rochester, Minnesota, United States; Mayo Clinic, Rochester, Minnesota, United States; Mayo Clinic, Rochester, Minnesota, United States

## Abstract

Tactile hallucinations can be highly distressing and lead to psychiatric morbidities and functional impairments. There are no established treatments for tactile hallucinations that occur outside of primary psychotic disorders, and available pharmacologic and nonpharmacologic therapies may have limited effectiveness. We present a case of a 75-year-old female with treatment-refractory tactile hallucinations successfully treated with electroconvulsive therapy (ECT). She endorsed 13 months of distressing tactile hallucinations described as slimly substances on her skin. Symptoms started insidiously, became more severe with time and refractory to multiple antipsychotic medications. She became so severely distressed from the tactile hallucinations that she developed a major depressive episode and became suicidal. Relevant medical history included one prior depressive episode, occipital lobe stroke, restless legs syndrome, and obstructive sleep apnea. She was hospitalized in an inpatient psychogeriatric unit and treated with ECT for 12 sessions over 3.5 weeks. Hallucinations responded well to ECT. Patient experienced some mild memory impairment from treatment. Treatment was optimized with duloxetine titration, addition of mirtazapine and repletion of B6 vitamin. Following the acute course of ECT, she reported an 80 % improvement in tactile hallucinations. Depressive symptoms improved, and suicidal thoughts resolved. She was able to discharge from the hospital and continued with maintenance ECT. Our case illustrates the potential therapeutic role of brain stimulation in the treatment of refractory cases of tactile hallucinations. Further studies are needed to determine effective treatment strategies for treatment-refractory tactile hallucinations to prevent progression to severe psychiatric disturbances and improve quality of life.

